# Holocene hydroclimatic variability in the tropical Pacific explained by changing ENSO diversity

**DOI:** 10.1038/s41467-022-34880-8

**Published:** 2022-11-25

**Authors:** Christina Karamperidou, Pedro N. DiNezio

**Affiliations:** 1 Department of Atmospheric Sciences, School of Ocean and Earth Science and Technology, University of Hawaii at Manoa, 2525 Correa Rd, HIG 350, Honolulu, HI 96815 USA; 2 Department of Atmospheric and Oceanic Sciences, University of Colorado Boulder, 311 UCB, Boulder, CO 80309 USA

**Keywords:** Palaeoclimate, Climate change

## Abstract

Understanding El Niño-Southern Oscillation (ENSO) response to past climate forcings is hindered by conflicting paleoclimate evidence. Records from the eastern Pacific show an intensification of ENSO variability from early to late Holocene, while records from the central Pacific show highly variable ENSO throughout the Holocene without an obvious relation to insolation forcing, which is the main climate driver during this interval. Here, we show via climate model simulations that conflicting Holocene records can be reconciled by considering changes in the relative frequency of the three preferred spatial patterns in which El Niño events occur (Eastern Pacific, Central Pacific, and Coastal) and in the strength of their hydroclimatic impacts. The relationship between ENSO diversity and variance is not only crucial for interpreting paleo-ENSO records and understanding ENSO response to external forcings but can also be used across climate model simulations to help evaluate the realism of ENSO projections in a changing climate.

## Abstract (web summary)

Past changes in climate variability across the tropical Pacific inferred from paleoclimate records can be explained by changes in both the frequency in which El Niño events with different spatial patterns occur, as well as their hydroclimatic impacts.

## Introduction

A substantial part of our understanding of externally-forced changes in the ENSO phenomenon has been shaped by rainfall-sensitive paleoclimate proxy records spanning the Holocene obtained from sites in the eastern equatorial Pacific, such as Peruvian mollusk records^1^ and lake sediment records from the tropical Andes (Laguna Pallcacocha^2,3^) and the Galapagos islands^4^. These records show much weaker hydroclimatic variability in the eastern tropical Pacific and coastal S. America in the early and middle Holocene and a significant strengthening towards the late Holocene, leading to the conclusion that ENSO is highly sensitive to changes of tropical Pacific climate driven by orbital variations. However, this view has been challenged by coral archives from the central Pacific whose isotopic composition, a quantity highly correlated with large-scale sea-surface temperature and salinity variability driven by ENSO events, remains highly variable throughout the Holocene without showing a discernible response to orbital forcing, but rather a step decrease of 30–70% compared to late twentieth century values^5–7^.

**Fig. 1:**
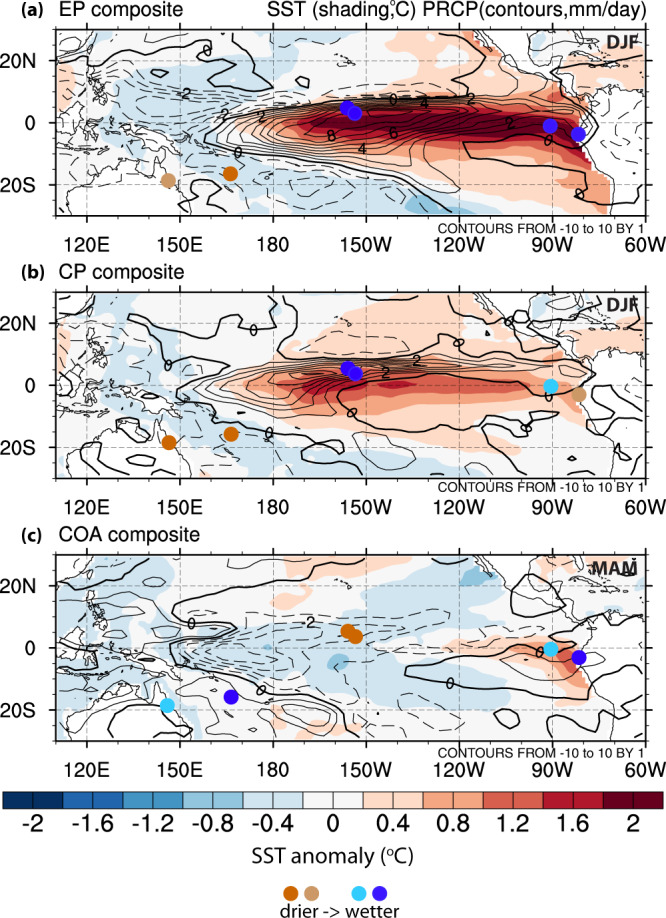
Observed hydroclimate impacts of El Niño-Southern Oscillation (ENSO) flavors. Composite sea-surface temperature (SST; shading) and precipitation (PRCP; contours) anomalies during the three peak months of **a** Eastern Pacific (EP), **b** Central Pacific (CP) and **c** Coastal (COA) El Niño events. The peak three months for EP and CP events are December-January-February (DJF), while for COA events they are March–April–May (MAM). Solid lines indicate positive precipitation anomalies, while dashed contours indicate a drying signal. Colored circles indicate characteristic locations of proxy records, with deeper colors indicating stronger precipitation response to each ENSO flavor (brown for drier, blue for wetter). The proxy locations shown are: The Great Barrier Reef [146.5 °E, 18.6 °S], Vanuatu [167.2 °E, 15.7 °S], Kiritimati [202.6 °E, 1.9 °N], Fanning [200.6 °E, 3.8 °N], Galapagos [270.3 °E,1.2 °S] and the Laguna Pallcacocha region in Ecuador [280.8 °E, 2.8 °S].

The seemingly contradictory changes seen in the eastern and central Pacific paleoclimatic records could be explained by changes in the frequency of ENSO “flavors”, also referred to as ENSO diversity, i.e. the different spatial patterns of sea-surface temperature anomalies during El Niño events^8^, and changes in their hydroclimatic impacts across the Pacific. Each flavor is associated with distinct sea-surface temperature (SST) and precipitation anomalies across the Pacific, sometimes of opposite sign (Fig. 1). Eastern Pacific (EP) events are accompanied by strong warm SST anomalies and an increase in precipitation in the eastern Pacific, while during Central Pacific (CP) events the SST anomalies there are weak and precipitation can be locally either increased or decreased (Fig. 1a, b). In the central Pacific, both EP and CP events induce SST and precipitation response of varying magnitude. During Coastal (COA) El Niño events, which peak in boreal spring (MAM), strong SST warming and increased precipitation are generally confined off of the coast of S. America, while basin-scale conditions remain in the neutral or even La Niña stage leading to cooler and drier conditions in the central Pacific and increased precipitation in the western Pacific (Fig. 1c). These large-scale patterns may change decadally or with changing background conditions^9–11^, and along with a change in the frequency and amplitude of the three flavors can ultimately be reflected in paleoclimate proxy records from across the Pacific. For example, a decrease in the frequency of Eastern Pacific (EP) and an increase in that of Central Pacific (CP) events, as has been shown in model simulations of the mid-Holocene^12^, can lead to compounded drier conditions in the eastern Pacific. Also, significant changes in the frequency of COA events can lead to substantial changes in rainfall variability in the far eastern Pacific due to their enhanced impact that is modulated by regional circulations and topography^13^.

Here, we assess the robustness of the “changing flavor” hypothesis^12^ for explaining tropical Pacific hydroclimatic changes during the Holocene by using climate simulations of key intervals performed with a global earth system model that simulates the features of all three ENSO flavors with significant realism^14^ (c.f Fig. 1 and Supplementary Fig. 1). Five simulations run under external forcings for 0, 3, 6, 9 and 12 thousand years before present (ka BP) represent four intervals throughout the Holocene with different climatic conditions driven mainly by the effect of orbital precession on seasonal insolation, with the exception of the 12 ka BP interval which includes ice sheet changes and lower greenhouse gases. These intervals allow us to resolve long-term trends in ENSO response driven by precessional forcing, while the simulations are sufficiently long (400–600 years each) to be able to discern forced changes in ENSO relative to the large unforced multi-decadal and centennial variability of the phenomenon^15^.

## Results and discussion

**Fig. 2:**
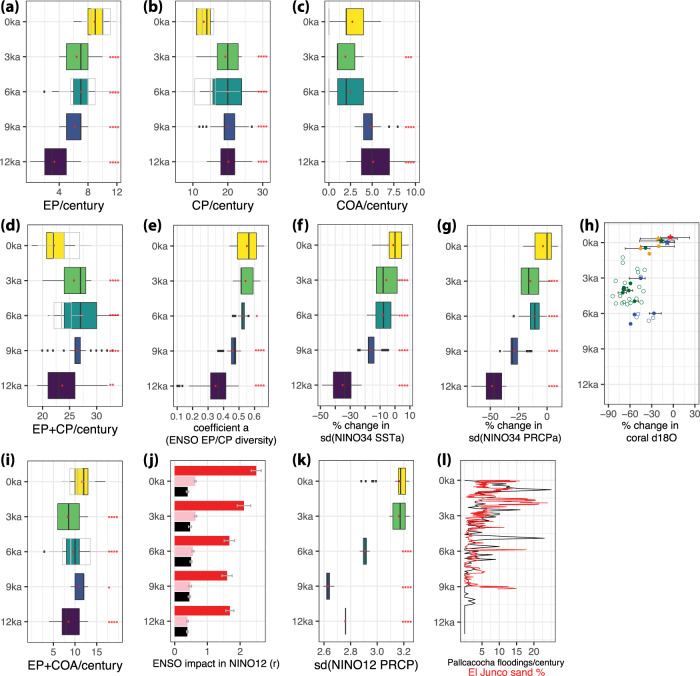
Simulated changes in El Niño-Southern Oscillation (ENSO) flavors in the Holocene (top), and simulated and proxy changes in central Pacific (middle) and far eastern Pacific (bottom) hydroclimate. The top panel shows the number of **a** Eastern Pacific (EP), **b** Central Pacific (CP), and **c** Coastal (COA) events per century in the Holocene time-slice experiments with the Community Earth System Model v1 (CESM1). The middle panel shows **d** the number of “central Pacific influence” (EP + CP) events per century, **e** the coefficient *α*, which is a metric of ENSO EP/CP diversity, the simulated changes in sea surface temperature (SST) (**f**) and precipitation (**g**) variability in the NINO3.4 region, and **h** the change in 1.5–10 yr standard deviation of coral *δ*^18^*O* from the central Pacific (Kiritimati-green^6^;Fanning-blue^5^;Palmyra-orange^26^) relative to the 1977–2007 reference period. In subfigure **h**, which is adapted from Karamperidou et al.^7^, stars indicate modern records (twentieth century), and circles show fossil coral records with open circles indicating records < 30 yr and error bars showing the range of 30 yr variability in coral records > 30 yrs. The bottom panel shows **i** the number of simulated “eastern Pacific influence” (EP + COA) events, **j** their impact in the far eastern Pacific as indicated by the regression coefficient between the December–January–February (DJF) EP (pink) and CP (black) index and March–April–May (MAM) COA index (red) and precipitation anomalies in the NINO1+2 region, **k** the standard deviation of total precipitation in the NINO1+2 region, and **l** the number of run-off events recorded in L. Pallcacocha^3^ and the percentage of sand in Lake El Junco (Gapalagos)^4^. Boxplots indicate the interquartile range (IQR) of values calculated from each simulation over one hundred random 100-yr samples. Black lines show the median, while whiskers show 1.5 times the IQR. The asterisks next to each boxplot indicate the significance in the change in means between the time-slice and the reference 0ka simulation based on a Wilcoxon test, as follows: *p ≤ 0.05, **p ≤ 0.01, ***p ≤ 0.001, ****p ≤ 0.0001. Gray boxplots show the number of events across 15 models participating in the Paleoclimate Model Intercomparison Project (PMIP) phase 3 & 4 for their pre-industrial control and mid-Holocene simulations.

In our simulations, the frequency of the three ENSO flavors shows consistent changes in response to the orbital forcing. We find a gradual increase in the frequency of EP events and a decrease in CP and COA events in the late Holocene compared to the early Holocene (Fig. 2). Across the Holocene experiments, the number of EP events is anti-correlated with the number of CP events with *r* = −0.68. These opposite changes (increase in EP and decrease in CP event frequency) are accompanied by an increase in the EP/CP ENSO diversity coefficient ∣*α*∣ (Fig. 2e and Supplementary Fig. 2) in the late Holocene. The decreased frequency of EP events in the early and middle Holocene is first due to a gradual deepening of the thermocline (Supplementary Fig. 3q–t) and decrease in eastern Pacific stratification (Supplementary Fig. 3l–o) during the development season of ENSO (JJASO), which leads to a weakening of the thermocline and upwelling feedbacks in the eastern Pacific (Supplementary Fig. 4a), and in turn an inhibition of the growth of EP events. In addition, the decrease in the east-west tropical Pacific zonal surface temperature gradient (apparent in Supplementary Fig. 3b–e) leads to a decrease in the zonal advection due to anomalous currents (*Q*_*ZA*,*curr*_; Supplementary Fig. 4a), which further contributes to weaker and less frequent EP events. On the contrary, the zonal advection due to anomalous currents increases in the NINO4 region towards the early Holocene, with the exception of the 12 ka simulation (Supplementary Fig. 4b). This allows CP event frequency to increase towards the early Holocene, even though it should be noted that this change comes primarily as a step change between present-day and 3ka rather than large gradual decreases like in EP events (Fig. 2b). These results support previous conclusions on the sensitivity of EP and CP ENSO to mid-Holocene orbital forcing^12,16,17^ and the significance of the thermocline/upwelling and zonal advective feedback for the EP and CP events, respectively^18^, although the relative importance of the different feedback terms in the evolution of ENSO flavors may depend on the model used. The higher frequency in simulated COA events in the early Holocene coincides with cooling of climatological SSTs during their peak season (March to June; see Supplementary Fig. 3b–e); such La-Niña-like mean state changes were found to be conducive to coastal warming by reducing atmospheric stability and destabilizing the ITCZ via atmospheric teleconnections^19^.

**Fig. 3:**
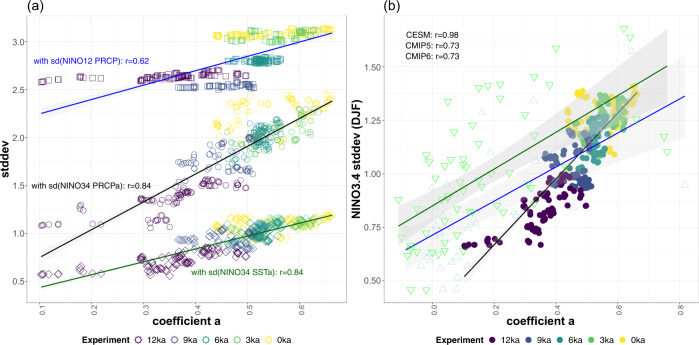
The relationship between El Niño-Southern Oscillation (ENSO) diversity and tropical Pacific sea-surface temperature (SST) and precipitation variability in pre-industrial control and paleoclimate model experiments. ENSO diversity is measured by the coefficient *α* of the quadratic fit in the December-January-February (DJF) PC1-PC2 plane; positive *α* indicates a concave down shape in the PC1–PC2 space, as in Supplementary Fig. 2. **a** Standard deviation of monthly hydroclimate variables in the central and eastern Pacific vs. ENSO diversity. Points correspond to 100-yr samples from each experiment with the Community Earth System Model v1 (CESM1), with their colors indicating the time slice and their shape indicating the variable plotted: diamonds and circles for the standard deviation of SST and precipitation anomalies in the central Pacific (NINO3.4 region), respectively, and squares for the standard deviation of total precipitation in the far eastern Pacific (NINO1+2 region). Shading indicates uncertainty in the regression lines. The adjusted R of a linear fit between *α* and each of the three variables is color-coded to match the regression lines and is reported next to each set of points (blue for the standard deviation of total NINO1+2 precipitation, black for the standard deviation of NINO3.4 precipitation anomalies, and dark green for the standard deviation of NINO3.4 SST anomalies). **b** DJF NINO3.4 standard deviation vs. ENSO diversity across past climate experiments with CESM1 and 40 CMIP5 & 62 CMIP6 models. Colored circles correspond to 100-yr samples from the CESM1 paleoclimate experiments, while open triangles show models participating in the Coupled Model Intecomparison Project (CMIP) 5 (blue, pointing up) and CMIP6 (green, pointing down). Shading indicates uncertainty in the regression lines (blue and green for CMIP5 and CMIP6 models, black for CESM1 paleoclimate experiments). The adjusted R of a linear fit in the three groups is reported at the upper left.

The relative frequency of EP and CP events changes in our simulations in a way consistent with the paleoclimatic evidence from the central Pacific. As shown in Fig. 1, EP and CP events impact precipitation and SST in the central Pacific (e.g., Kiritimati and Fanning), with EP events exerting a stronger influence in SST compared to CP events both in observations and in the model (cf. Fig 1 and Supplementary Fig. 1). There is a step increase in the sum of these “central Pacific influence events” (EP + CP) between 0 ka and 3 ka, which continues in the mid-Holocene (Fig. 2d). However, the number of EP and CP events is highly variable during the Holocene, with their median frequency ranging from 22 to 27 events per century, as shown in Fig. 2d. Importantly, it is their relative frequency -or in other words the ENSO EP/CP diversity (Fig 2e)- rather than their sum that most strongly influences the precipitation response across the Holocene. The decrease in EP events (Fig. 2a) and increase in CP events (Fig. 2b) translates into a decrease in the coefficient *α* of ENSO diversity in the early Holocene (Fig. 2e) that is mostly significant in 9 ka and 12 ka. This decrease is consistent with the simulated decreases in SST and precipitation variability towards the early Holocene (9 ka, 12 ka) in the central Pacific seen in Fig. 2f, g because the relative impact of EP events in the region is stronger compared to that of CP events (Fig. 1a, b). The impact of the relative frequency of the two dominant ENSO flavors is also seen in the positive correlation between the metric of ENSO EP/CP diversity (coefficient *α*) and the standard deviation of SST and precipitation anomalies in the central Pacific (Fig. 3a): Smaller *α* indicates fewer EP events compared to CP events, which is associated with less SST and precipitation variability in the NINO3.4 region. Thus, it is clear that changes in the ENSO EP/CP diversity throughout the Holocene can explain the variability in central Pacific SST and precipitation: the general stability in *α* between 3 ka and 6 ka in Figs. 2e and 3a agrees with the lack of significant changes in the simulated SST and precipitation variability (Fig. 2f, g) and with the central Pacific proxy records^5,6^, which show highly variable ENSO activity during the last 6000 years with the exception of the modern twentieth century records (Fig. 2h).

In the eastern Pacific, EP and COA events positively impact precipitation, while CP events are responsible for a weak wet and even drying signal in parts of coastal S. America (Fig. 1). The sum of the “eastern Pacific influence events” (EP + COA) decreases from a median number of 12 events per century in present-day climate to 8 events in early Holocene (Fig. 2i), amounting to an average early Holocene decrease of about 37.5%. In addition to the decrease in EP+COA event frequency, their impact is approximately 40% weaker in 12ka compared to 0ka (Fig. 2j), as expressed by the regression coefficients between precipitation anomalies in the far eastern Pacific and the NINO12res and E-index in boreal spring and winter, respectively. The decrease in eastern Pacific events and concurrent increase in CP event frequency going back to the early Holocene is reflected in the decrease of coefficient *α* (weaker ENSO EP/CP diversity), which is correlated with the standard deviation of total precipitation in the far eastern Pacific, as shown in Fig. 3a. Therefore, a decrease in ENSO EP/CP diversity and in the frequency of “eastern Pacific influence” events (EP+COA) along with a significant weakening of their influence lead to a compounded drying signal, which is consistent with the simulated lower precipitation variability over the NINO1+2 region (Fig. 2k) in the early Holocene (2.6–2.8 mm/day in 9 and 12 ka) compared to present-day (3.2 mm/day). This can help better explain the dramatic decrease in run-off events shown in the L. Pallcacocha and L. El Junco proxy records^3,4^ in the early Holocene (Fig. 2l). The timing of the simulated changes in rainfall variability whose biggest steps occur from 3ka to the mid-Holocene to 9 ka (Fig. 2k) is also in agreement with the decrease in the number of recorded run-off events in the lake sediments in the eastern Pacific in mid- to early Holocene (Fig. 2l).

**Fig. 4:**
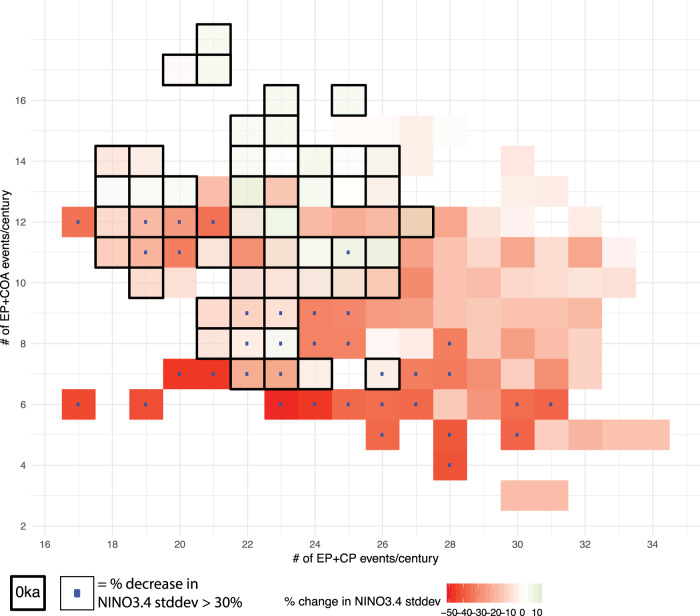
Simulated relative changes in El Niño-Southern Oscillation (ENSO) flavors in the Holocene. Number of “eastern Pacific influence” events (Eastern Pacific (EP) + Coastal (COA); ordinate) vs number of “central Pacific influence” events (Eastern Pacific (EP) + Central Pacific (CP); abscissa) in time-slice Holocene experiments with the Community Earth System Model v1 (CESM1). Each tile represents 100 yr samples to account for internal variability. Colored shading shows the percentage change in the standard deviation of NINO3.4 SST anomalies compared to the pre-industrial control simulation (0ka). Black-delineated tiles show 100-yr samples from the 0ka simulation. Blue dots indicate all 100-yr samples where NINO3.4 standard deviation decreases more than 30% compared to 0 ka. Note that the color shading corresponds to the 0ka values when 0ka tiles overlap with tiles from other Holocene experiments; the same combination of event frequency may lead to a >30% decrease in NINO3.4 standard deviation even though the color in the forefront may show a different value from the 0ka experiment (e.g. at the (26,7) tile).

We have thus showed that from the relative changes in the number of the EP, CP and COA events during the Holocene, it would be expected to see a highly variable hydroclimate in the central Pacific between mid- and late Holocene, but an intensification of precipitation variability in the eastern Pacific from the early to late Holocene. Disregarding ENSO diversity and using single indices, such as NINO3.4, to characterize ENSO activity and interpret paleoclimate proxy records can therefore be misleading. To further demonstrate this, we show in Fig. 4 how the frequency of EP, CP and COA events translates into changes in the NINO3.4 Index variance across all CESM1 Holocene experiments. Within the pre-industrial control simulation (0 ka), there exist multiple combinations of the number of “central Pacific influence events” (EP + CP) and “eastern Pacific influence events” (EP + COA) that can lead to either an increase or a decrease of NINO3.4 standard deviation, by as much as ±15% compared to the average NINO3.4 standard deviation of the simulation. For example, in a given century, a sum of 12 EP and COA events and a sum of 19 EP and CP events translates to 15% decrease of NINO3.4 standard deviation compared to 0ka. But the same decrease can be achieved by a sum of 7 EP and COA events and a sum of 22 EP and CP events, or a sum of 5 EP + COA and a sum of 32 EP + CP events. As became evident from the results presented in this study, even though the NINO3.4 standard deviation is the same across these three scenarios, the fact that it stems from vastly different combinations of EP, CP and COA events would lead to different hydroclimate manifestations in the same location, given the different impacts of these ENSO flavors.

Proxy records from the central tropical Pacific show decreases in ENSO standard deviation from 30 to about 70% compared to late twentieth century values^5–7^ (Fig. 2h). As shown by the blue dots in Fig. 4, reduction larger than 30% in NINO3.4 standard deviation can correspond to a wide range of frequencies of EP, CP and COA events: from cases with increased frequency of EP and COA events but strong reduction in the frequency of CP events, to cases where the exact opposite is true, i.e. CP events dominate. In addition, short coral records may be inadvertently subsampling any tile in Fig. 4, or in other words, any random 100-yr period during each Holocene time-slice. Thus, proxy-inferred changes in SST or precipitation variability need not necessarily reflect externally-forced changes in the frequency of ENSO events, and vice versa.

The complex manifestation of the relative frequency of ENSO flavors onto SST and precipitation variability across the Pacific raises the question, what is the relationship between ENSO variance -generally characterized by the variance of a single index such as NINO3.4- and ENSO diversity. Across the Holocene paleoclimate CESM1 experiments (Fig. 3b), the stronger the diversity (coefficient *α*), the stronger the DJF NINO3.4 standard deviation (DJF). Note that this is not necessarily expected, since strong NINO3.4 variance can be the result of strong activity in a single ENSO “flavor”^20^. Importantly, the linear ENSO diversity-variance relationship in the CESM1 experiments (*r* = 0.98) closely resembles the relationship across the pre-industrial control experiments with both CMIP5 and CMIP6 models (triangles in Fig. 3b; *r* = 0.73). The similarity in this relationship between CESM1 paleoclimate experiments and CMIP5 and 6 models suggests analogies between past climate changes and mean climate biases in models, which can be used to constrain model uncertainty in future climate projections. It should be noted that more than half of the models in both cohorts do not exhibit strong ENSO diversity/nonlinearity, as was also found in previous studies^12,21^ and seen in Fig. 3b. The ENSO diversity and variance relationship breaks down in these less skillful models where stronger NINO3.4 variance does not correlate with the presence of two ENSO flavors; this has consequent implications for faithful representation of global ENSO impacts as the latter are significantly different between flavors (Fig. 1; also see Taschetto et al.^11^ for a review).

In conclusion, interpretation of hydroclimatic changes in proxy records needs to take into account changes in the frequency of all ENSO flavors, their combinations and relative frequency, as well as changes in the strength of their impacts. In this study, we used a suite of time-slice earth system model simulations of multiple intervals since 12 ka BP to show that combined changes in the frequency of Eastern Pacific, Central Pacific, and coastal El Niño events, as well as a change in the strength of the hydroclimate impact of these flavors, reconcile central Pacific proxies that show highly variable ENSO in the Holocene with eastern Pacific proxies indicating large decreases in ENSO-related hydroclimatic variability in the early Holocene. In addition, a seeming contrast between a central or western Pacific proxy record indicating enhanced La Niña conditions and an eastern Pacific proxy indicating enhanced El Niño conditions over the same past period can be reconciled as partially resulting from increased frequency of Coastal events instead of a change in basin-scale ENSO, due to the fact that Coastal El Niño events have supersized precipitation impacts in the far eastern Pacific^13,22,23^ during otherwise basin-scale neutral or La Niña conditions. Our focus on the impacts of changes in the frequency and impacts of all ENSO flavors can thus help frame and better interpret past or future response of ENSO to other external forcings, including greenhouse gas emissions and volcanic eruptions, and its global and tropical hydroclimatic impacts, as clearly shown by the strong correlation between ENSO diversity and SST and precipitation variability in both the central and eastern Pacific.

As a broader implication of our study, we showed that the relationship of ENSO variance and EP/CP ENSO diversity in the paleoclimate CESM1 experiments spans the range of this relationship across the CMIP5/6 pre-industrial control experiments. This means that it is possible that model biases in simulating annual mean and seasonal climate of the tropical Pacific that resemble the forced changes across paleoclimate simulations can be linked to model disagreement with respect to ENSO variance and diversity and their future projections. Such connections can increase the robustness of previous studies^20,21^ that have used metrics of model skill in simulating ENSO diversity to classify models with respect to their projections of future changes in the mean tropical Pacific climate and ENSO variability itself.

## Methods

### Observations and proxy records

To calculate observed ENSO flavors and their impacts we use the HadISSTv4^24^ and GPCP^25^ datasets. The proxy records shown here are run-off events in sediment cores of L. Pallacocha^3^, percentage of sand in sediment cores of L. El Junco^4^, and isotope records (*δ*^18^*O*) from modern and fossil corals^5,6,26^.

### Model simulations

To assess the response of ENSO flavors to orbital forcing over the past 12,000 years (12 ka), we use a suite of time-slice experiments in 3 ka intervals with version 1 of the Community Earth System Model (CESM1)^14^. Each experiment is 400–600 years long and was run until the surface climate and oceanic processes controlling tropical climate, such as the depth of the thermocline in the equatorial Pacific or the Atlantic Meridional Overturning Circulation (AMOC), have reached equilibrium. All simulations exhibit minimal drift in global mean surface temperature (less than 0.05 °C per century), tropical mean surface temperature (less than 0.04 °C per century), the depth of the equatorial thermocline in the Pacific (less than 0.3 m per century), and the strength of the AMOC (less than 0.25 Sv per century) during the periods used in the analyses. With the exception of the 12 ka BP interval which includes ice sheet changes and lower greenhouse gases, the primary forcing in the 0, 3, 6, and 9 ka BP intervals is changes in Earth’s precession, and each simulation branched off its preceding one, starting from 0ka sequentially through the Holocene. The maximum TOA energetic imbalance does not exceed 0.45 Wm^−2^, which is much smaller than the imposed radiative forcing. CESM1 is one of the “better” models in terms of simulation of ENSO and its diversity^27,28^. The model is able to reasonably capture the frequency and patterns of EP, CP and COA events, and their impacts on precipitation (cf. Fig. 1 and Supplementary Fig. 1). The strength of EP and CP variability in the model’s control simulation (0 ka) is approximately equal to the ensemble mean of the CMIP5 and 6 model cohorts^29^. Even though the model overestimates ENSO variance as measured by the DJF NINO3.4 standard deviation by 6–20% compared to 20th century sea-surface temperature reconstructions, it simulates 8-10 EP and 11–16 CP events per century, which represents a realistic CP/EP-event ratio of 1.6. Using the same method^30^ for identifying EP and CP events, the CP/EP ratio since 1950 is 1.8^8^, while Freund et al.^31^ found a CP/EP ratio of 37/27 = 1.4 using paleoclimate proxy reconstructions over the period 1617–1920. The number of EP and CP events per century in the 0 ka and 6 ka CESM1 simulations is also comparable to that in 15 PMIP3/4 models (Fig. 2a, b), however, it should be noted that only 5 of the 15 models have a coefficient alpha (α; see definition in the following section) that indicates concavity in the PC1-PC2 space and that is significantly different from zero as in observations and CESM1. The heat budget analysis in the equatorial strip uses the methodology of DiNezio & Deser^32^ but with the mixed-layer depth being constant in time but variable in space (See Supplementary Information for a heat budget discussion).

### ENSO diversity

An EP event is defined in the model simulations when the Oct–Apr E-index exceeds 1.5 standard deviations; a CP event is defined when the C-index exceeds one standard deviation (indices defined by Takahashi et al.^30^). To identify COA events we use the NINO12res index, which represents the residuals of the linear regression of the NINO1+2 index onto the NINO3.4 index, thereby removing the linear ENSO signal associated with basin-scale events^33^. A COA event is defined when (1) the NINO12res index exceeds one standard deviation for at least three consecutive months between March and June, and (2) the concurrent NINO3.4 index is less or equal to 1/3 of its standard deviation. These events peak in boreal spring, therefore coinciding with the season of peak climatological rainfall in equatorial S. America. To characterize EP/CP ENSO pattern diversity in model simulations, we use the coefficient *a* of a quadratic fit in the phase plane of the first and second principal components of DJF SST anomalies in the tropical Pacific (10 °S–10 °N), proposed by Karamperidou et al.^20^ and shown in Supplementary Fig. 2. Since coefficient *α* is calculated based on the DJF SST anomalies, it excludes COA events, which peak in boreal spring and summer and do not have large-scale global impacts. Here, positive *a* indicates a concave down shape in the PC1–PC2 space, as in Supplementary Fig. 1.

We use linear regression coefficients between monthly SST and precipitation anomalies and the E, C and NINO1+2res indices to show impact changes across climates. To illustrate the differing impacts of EP, CP and COA events, we highlight six proxy locations (Fig. 1), as follows. Western Pacific: The Great Barrier Reef [146.5 °E, 18.6 °S] and Vanuatu [167.2 °E, 15.7 °S]^34,35^; central Pacific: Kiritimati [202.6 °E, 1.9 °N] and Fanning [200.6 °E, 3.8 °N]^5,6,36,37^; eastern Pacific: Galapagos [270.3 °E, 1.2 °S] and the Laguna Pallcacocha region in Ecuador [280.8 °E, 2.8 °S]^2–4,38–40^.

To account for internal climate variability in each time-slice simulation, all metrics or indices are computed from one hundred random 100-yr long samples, and the results are given as boxplots or tiles in the figures. The boxplots show the interquantile range (IQR) from these 100 samples from each simulation, while whiskers indicate 1.5 times the IQR. To compare means between time-slices and the reference 0ka simulation, we apply a Wilcoxon test (non-parametric). The sampling size (i.e. 100 years) is chosen as a compromise between typical lengths of instrumental, coral and lower resolution proxy records, as well as to correspond to the reporting window for proxy-inferred event frequency (e.g., in Moy et al.^3^).

## Supplementary information

Supplementary Information

## Data Availability

All published coral and lake sediment records are publicly available via the NOAA National Centers for Environmental Information paleoclimatology archives at https://www.ncei.noaa.gov/products/paleoclimatology. Observational datasets (HadISSTv4 and GPCP) are available via the respective authoritative sites (https://www.metoffice.gov.uk/hadobs/hadisst/ and https://www.ncei.noaa.gov/products/global-precipitation-climatology-project). All PMIP3/4 and CMIP5/6 data are publicly available through the Earth System Grid Federation datanodes at https://esgf-node.llnl.gov/projects/esgf-llnl/. The model output and source data used in this study are available on Zenodo 10.5281/zenodo.7302480.

## Code availability

This article uses the languages R (https://www.R-project.org/) and NCL (https://www.ncl.ucar.edu/) to perform the reported analyses; codes can be accessed upon request to C.K. For the analysis, we used the computing resources provided by the University of Hawaii at Manoa and NCAR’s Computational and Information Systems Laboratory, sponsored by the National Science Foundation^41^.
