# Supplementary material for: Holocene hydroclimatic variability in the tropical Pacific explained by changing ENSO diversity
Source: Nat Commun. 2022 Nov 25;13:7244. doi: 10.1038/s41467-022-34880-8 (PMC9700811; doi:10.1038/s41467-022-34880-8)
Supplement: Supplementary file 1 — Supplementary Information [file 41467_2022_34880_MOESM1_ESM.pdf]

Supplementary Information for:

Holocene hydroclimatic variability in the tropical Pacific explained by changing ENSO diversity.

Christina Karamperidou[1*]
& Pedro N. DiNezio[2]

[1]Department of Atmospheric Sciences, School of Ocean and Earth Science and Technology
2525 Correa Rd, Honolulu, HI 96815, USA

[2]Department of Atmospheric and Oceanic Sciences, University of Colorado Boulder, 311 UCB, Boulder, CO 80309, USA

[*]Correspondence to: ckaramp@hawaii.edu

This PDF file includes:

- Head budget discussion
- Supplementary Figures 1 – 5

Heat budget discussion

The methodology used to estimate the terms of the temperature equation follows DiNezio & Deser (2014). The full heat budget can be approximated by

$$\rho_0 c_p H \frac{\partial [T']}{\partial t} \cong -\rho_0 c_p \int_{-H}^{0} \left(u' \frac{\partial \overline{T}}{\partial x} + \overline{u} \frac{\partial T'}{\partial x} + u' \frac{\partial T'}{\partial x} + w' \frac{\partial \overline{T}}{\partial z} + \overline{w} \frac{\partial T'}{\partial z} + w' \frac{\partial T'}{\partial z} \right) dz + Q'_{atm} \tag{1}$$

where primed variables are anomalies with respect to the climatological monthly-mean seasonal cycle indicated by overbar variables. Meridional advection and the meridional nonlinear terms are neglected because their interannual variability is small. The variables are integrated over a mixed-layer depth H which is constant in time but variable in space.

The thermocline term is the anomalous heat flux convergence associated with the advection of temperature anomalies (anomalous thermocline) by the climatological upwelling:

$$Q'_{TC} = -\rho_0 c_p \int_{-H}^{0} \overline{w} \frac{\partial T'}{\partial z} \, dz \tag{2}$$

The upwelling term is the anomalous heat flux convergence associated with the advection of the vertical climatological temperature gradient by anomalous upwelling:

$$Q'_{UW} = -\rho_0 c_p \int_{-H}^{0} w' \frac{\partial \overline{T}}{\partial z} \, dz \tag{3}$$

The zonal advection term associated with the advection of the zonal climatological temperature gradient by anomalous zonal currents is given by:

$$Q'_{ZA,curr} = -\rho_0 c_p \int_{-H}^{0} u' \frac{\partial \overline{T}}{\partial x} \, dz \tag{4}$$

while the zonal advection term associated with the advection of temperature anomalies from the climatological zonal current:

$$Q'_{ZA,temp} = -\rho_0 c_p \int_{-H}^{0} \overline{u} \frac{\partial T'}{\partial x} \, dz \tag{5}$$

The nonlinear zonal and vertical advection terms are given by $Q'_{u_{nl}} = -\rho_0 c_p \int_{-H}^{0} u' \frac{\partial T'}{\partial x}\, dz$ and $Q'_{w_{nl}} = -\rho_0 c_p \int_{-H}^{0} w' \frac{\partial T'}{\partial z}\, dz$.

To show the relative contribution of each term of the heat budget to the evolution of EP and CP events across the simulated climates, we composite their values for $\pm$ 16 months from the peak of the events in the NINO3 and NINO4 regions, respectively, and smooth with a loess filter.

In Supp. Fig. 3 we show the climatological changes in the surface ocean temperature (tos), zonal current (uos), stratification (DTml) and thermocline depth (ztc). The climatological changes in the surface temperature and resulting zonal temperature gradient will affect the zonal advection due to anomalous currents ($Q'_{ZA,curr}$), while the changes in the climatological zonal current will affect the $Q'_{ZA,temp}$ term, and changes in stratification will affect the Q'_{UW} term.

For EP events we focus in the NINO3 region where the anomalies are peak (Supp. Fig. 4a). The advection of the climatological temperature gradient by anomalous upwelling ($Q'_{UW} = -\rho_0 c_p \int_{-H}^{0} w' \frac{\partial \overline{T}}{\partial z}\, dz$), the advection of temperature anomalies (anomalous thermocline) by the climatological upwelling ($Q'_{TC} = -\rho_0 c_p \int_{-H}^{0} \overline{w} \frac{\partial T'}{\partial z}\, dz$), and the zonal advection by anomalous currents ($Q'_{ZA_{curr}} = -\rho_0 c_p \int_{-H}^{0} u' \frac{\partial \overline{T}}{\partial x}\, dz$) act to enhance positive SST anomalies, while the non-linear zonal ($Q'_{u_{nl}} = -\rho_0 c_p \int_{-H}^{0} u' \frac{\partial T'}{\partial x}\, dz$) and vertical ($Q'_{w_{nl}} = -\rho_0 c_p \int_{-H}^{0} w' \frac{\partial T'}{\partial z}\, dz$) advection terms act as negative feedbacks. The advection of temperature anomalies by the climatological zonal current ($Q'_{ZA,temp} = -\rho_0 c_p \int_{-H}^{0} \overline{u} \frac{\partial T'}{\partial x}\, dz$) plays a very small role. The primary change in the evolution of EP events as we move back towards the early Holocene (12ka) is a decrease in the contribution of the zonal advection by anomalous currents ($Q_{ZA,curr}$), as well as in the contribution of the thermocline and upwelling terms (Q'_{TC}, Q'_{UW}). The nonlinear zonal advection term becomes a small positive heat flux contribution in early Holocene, from being a negative contribution in present-day climate. The vertical nonlinear advection is a negative contribution across the simulations, but becomes smaller in magnitude towards the early Holocene (12ka).

For CP events, we focus in the NINO4 region, where we find that the main contribution to the evolution of the events is the zonal advection by anomalous currents ($Q_{ZA,curr}$), while the thermocline term and the advection of temperature anomalies by the climatological currents have smaller contributions. Zonal advection by anomalous currents increases towards the early Holocene, except in the 12ka simulations, which is consistent with the increased CP frequency of the paleo-simulations compared to present day albeit with relatively small changes between 3ka and 12ka.

Note that the peak of the above composites is based on the peak magnitudes of the E-index and C-index, and thus the peak of SST anomalies in each NINO region may not coincide exactly with the peak of the indices. Also note that the composites are averaged using the observed ENSO regions: [5°S-5°N, 150°W-90°W] for NINO3, and [5°S-5°N, 160°E-150°W] for NINO4.

Supplementary Figures

- **Supplementary Figure 1:** Simulation of El Niño-Southern Oscillation (ENSO) flavors in the Community Earth System Model v1.2 (CESM1.2).
- **Supplementary Figure 2:** ENSO EP/CP diversity in Holocene simulations with the Community Earth System Model v1.2 (CESM1.2).
- **Supplementary Figure 3:** Climatological changes in Holocene simulations with the Community Earth System Model v1.2 (CESM1.2).
- **Supplementary Figure 4:** Composite heat budget during the development, peak, and decay of Eastern Pacific (EP) and Central Pacific (CP) events.
- **Supplementary Figure 5:** The relationship between simulated NINO3.4 standard deviation and the standard deviation of monthly total precipitation in the eastern Pacific and

monthly precipitation anomalies in the central Pacific.

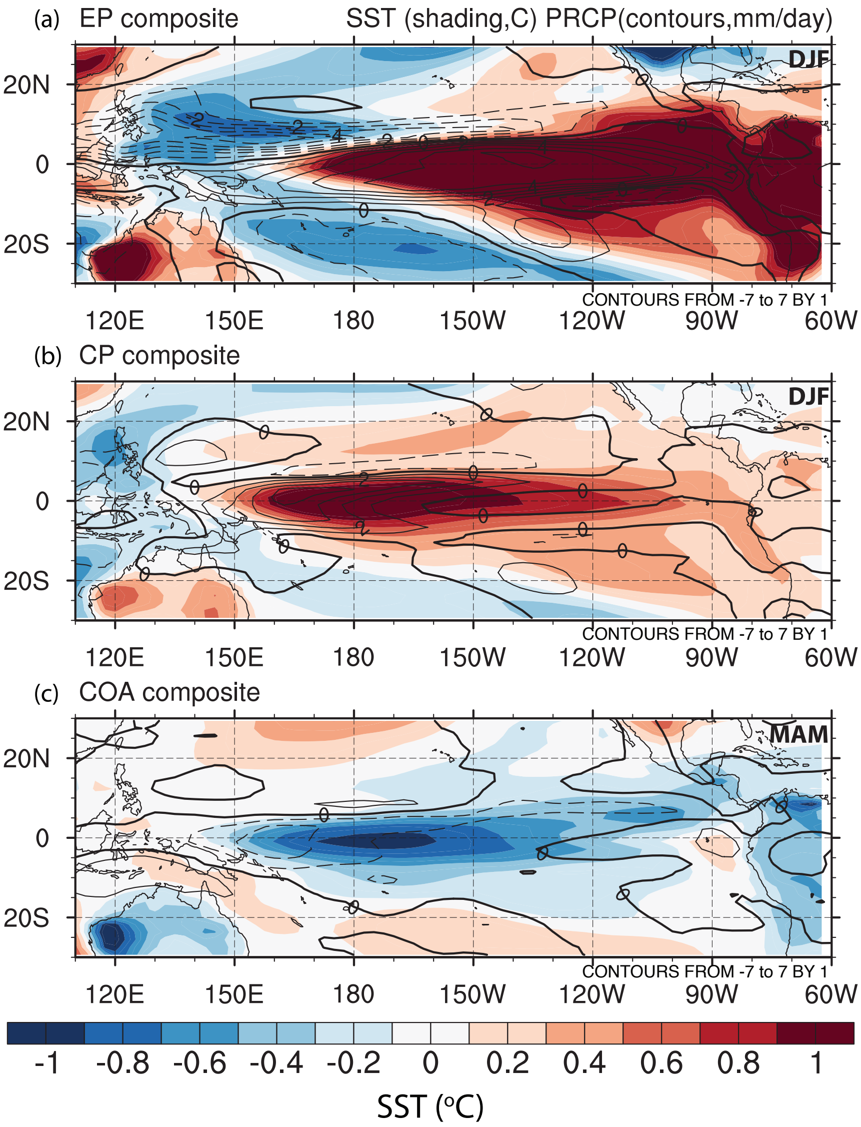

Figure 1: Simulation of El Niño-Southern Oscillation (ENSO) flavors in the Community Earth System Model v1.2 (CESM1.2). Composite sea surface temperature (SST; shading) and precipitation (PRCP; contours) during the three peak months of a) Eastern Pacific (EP), b) Central Pacific (CP) and c) Coastal (COA) El Niño events. All index and regression calculations are as in Fig. 1 of the main article, using output from CESM1.2 pre-industrial control simulation (0ka). The peak three months for EP and CP events are December-January-February (DJF), while for COA events they are March-April-May (MAM).

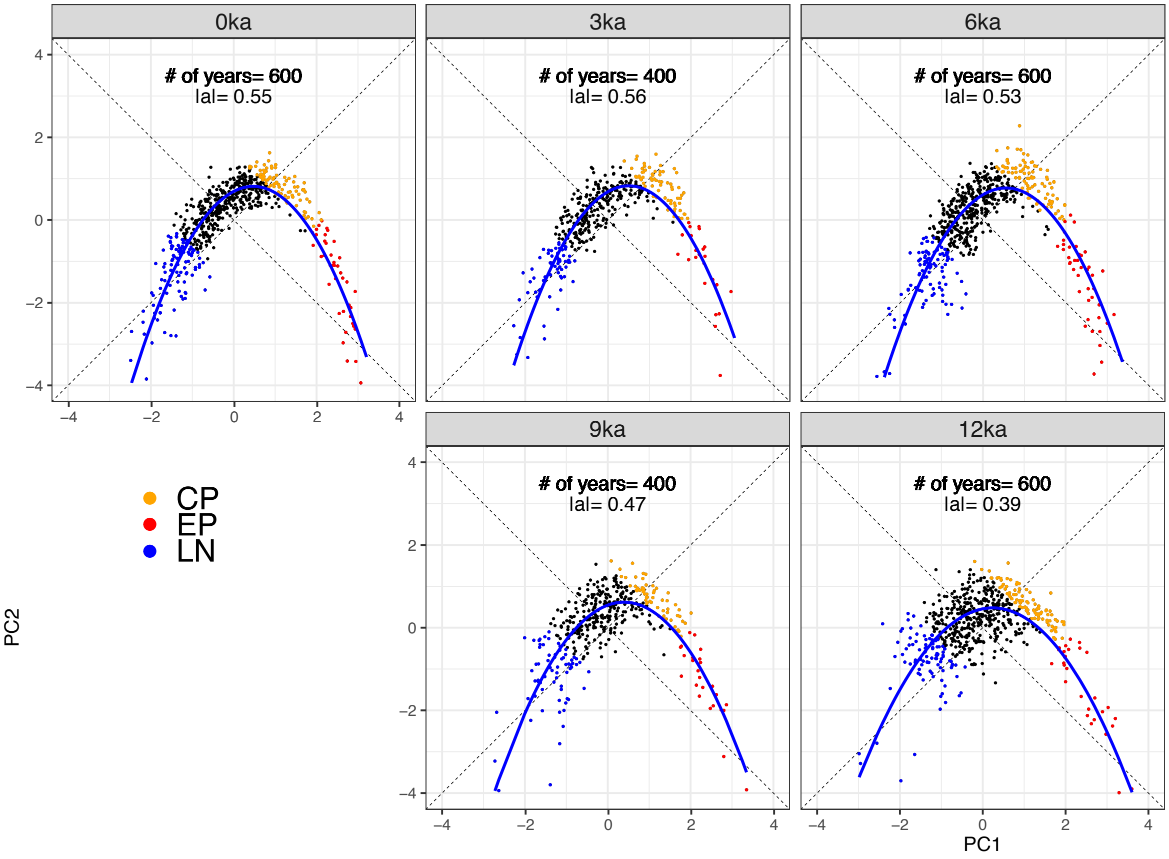

Figure 2: ENSO EP/CP diversity in Holocene simulations with the Community Earth System Model v1.2 (CESM1.2). Scatterplots of the first and second principal components of December-January-February (DJF) SST anomalies in the tropical Pacific (PC1 and PC2) in the CESM1.2 control experiment (0ka) and the Holocene time-slice simulations (3,6,9, and 12ka). Orange, red and blue colors indicate Central Pacific (CP), Eastern Pacific (EP) and La Niña events, as defined in the methods. Fitted quadratic curves measure ENSO EP/CP diversity via their coefficient alpha (α) reported in each figure. The EOF analysis is performed in the tropical Pacific bounded latitudinally by 10° S–10°N.

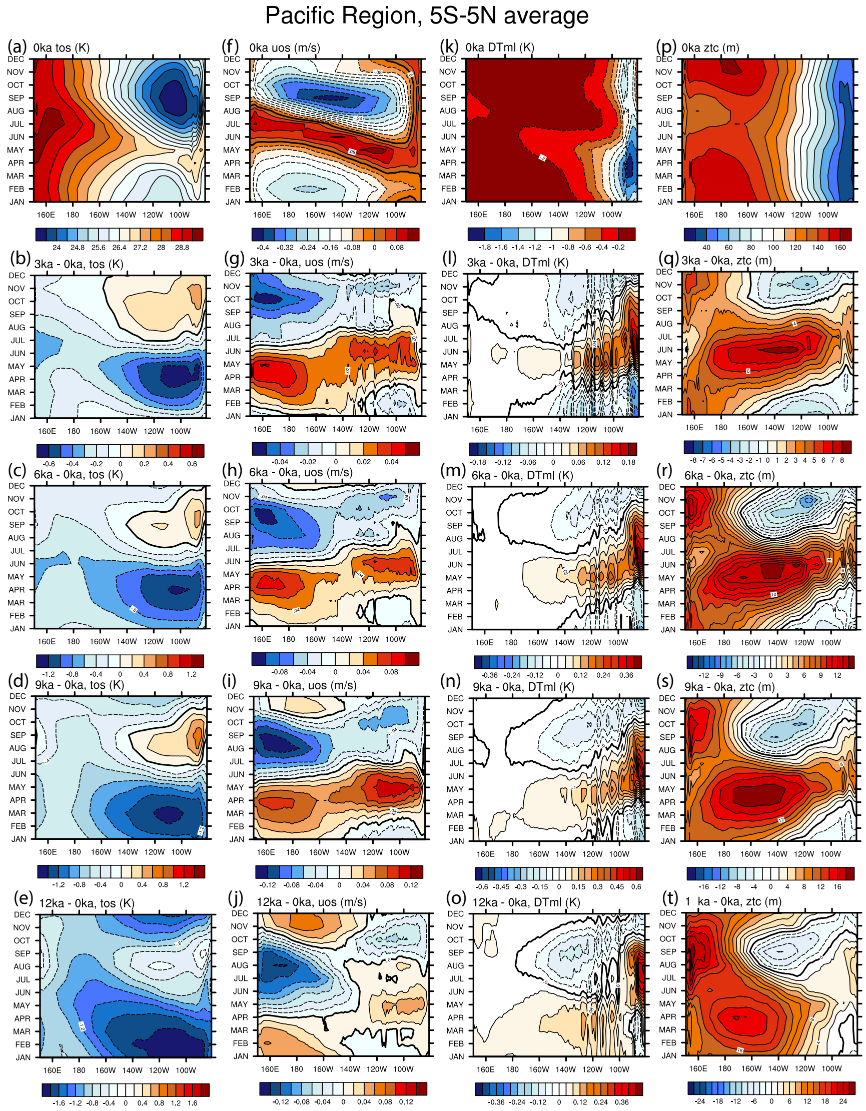

Figure 3: Climatological changes in Holocene simulations with the Community Earth System Model v1.2 (CESM1.2). (left to right panels) Time-longitude plots of climatological surface ocean temperature (tos), surface ocean currect velocity (uos), stratification in the mixed layer (DTml) and thermocline depth (ztc) in the model's control simulation (0ka; top row), and their difference with the Holocene time-slice simulations (3,6,9, and 12ka). Stratification is defined as Tsub - Ts, where Ts is the surface temperature and Tsub is the temperature at depth of 50m; a positive difference indicates warming of the subsurface, i.e. a decrease in stratification. All fields are averaged between $5°S$ and $5°N$.

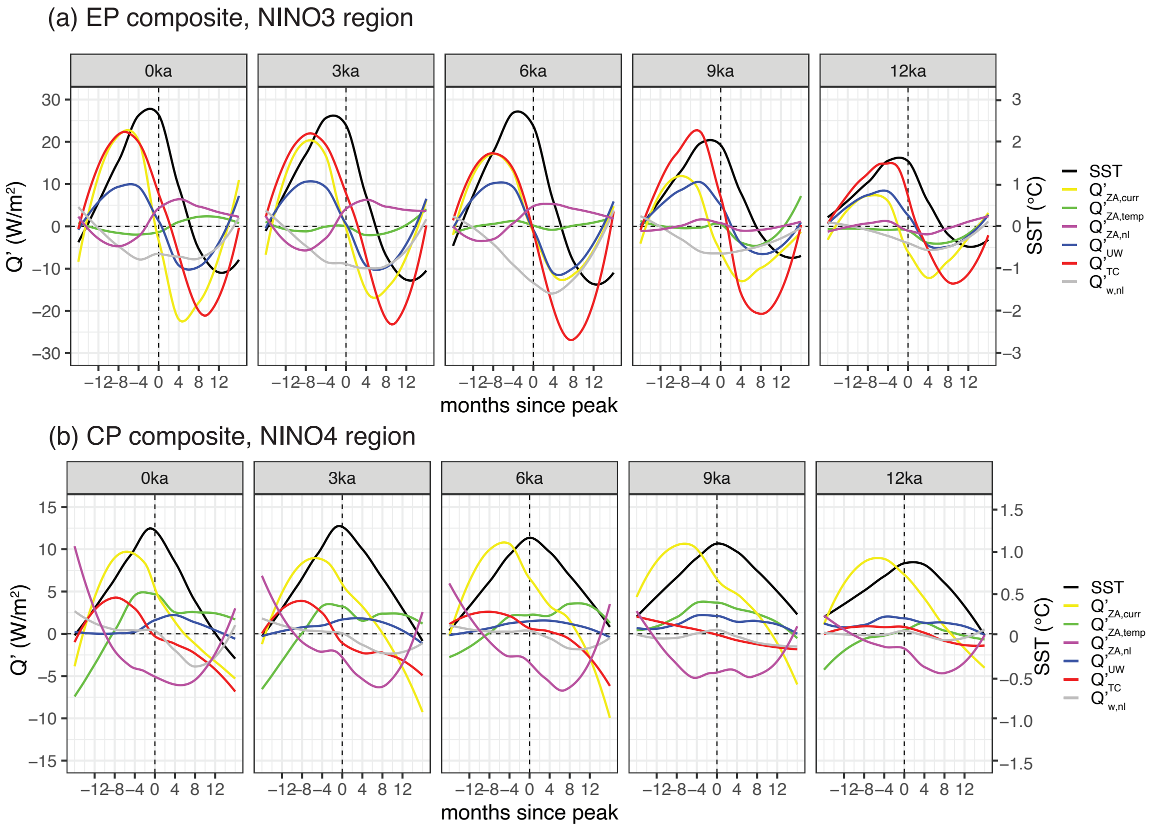

Figure 4: Composite heat budget during the development, peak, and decay of (a) Eastern Pacific (EP) events in the NINO3 region, and (b) Central Pacific (CP) events in the NINO4 region. Black lines show the sea surface temperature (SST) anomaly, while colored lines show the ocean dynamical heating: zonal advection due to anomalous currents (yellow; $Q_{ZA,curr}$), zonal advection by climatological currents (green; $Q_{ZA,temp}$), nonlinear zonal advection (magenta; $Q_{ZA,nl}$), vertical advection due to anomalous upwelling (blue; Q_{UW}), vertical advection of thermocline anomalies (red; Q_{TC}), and nonlinear vertical advection (grey; $Q_{w,nl}$. Positive values of heating terms indicate a warming tendency. The composites are smoothed with a loess filter.

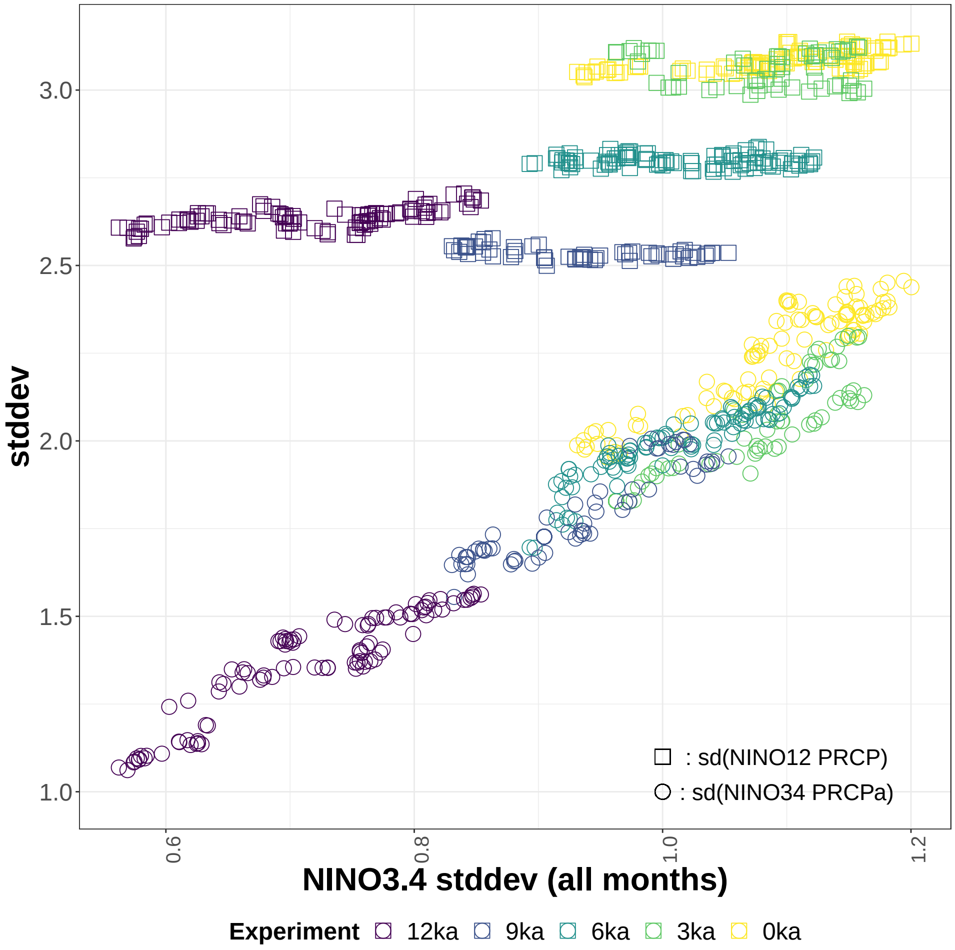

Figure 5: The relationship between simulated NINO3.4 standard deviation and the standard deviation of monthly total precipitation in the eastern Pacific (NINO1.2 region; squares) and monthly precipitation anomalies in the central Pacific (NINO3.4 region; circles). Points correspond to 100-yr samples from each experiment, with their colors indicating the time slice and their shape indicating the variable plotted.
